# Tuberous Sclerosis Due to Deletion of Exons 4–8 in TSC2 Gene, Favourably Responding to Phenytoin and Everolimus: A Case Report

**DOI:** 10.7759/cureus.79764

**Published:** 2025-02-27

**Authors:** Josef Finsterer

**Affiliations:** 1 Neurology, Neurology and Neurophysiology Center, Vienna, AUT

**Keywords:** adjustment disorder, anxiety disorder, a patient with tuberous sclerosis (tsc) due to a previously unknown mutation, autism spectrum disorder, claustrophobia, developmental delay, mild cognitive impairment, multiple hamartomas throughout the body, recurrent depressive episodes

## Abstract

A case of tuberous sclerosis (TSC) caused by a previously unreported mutation, with epilepsy responding well to phenytoin (PHT) and multilocular benign tumors regressing with everolimus, has not been documented before, to the best of our knowledge. The patient is a 24-year-old female who was diagnosed with tuberous sclerosis complex (TSC) at the age of seven due to epilepsy and the presence of multiple hamartomas, including angiofibromas, angiomyolipomas, lymphangioleiomyomas, rhabdomyomas, and astrocytomas, throughout her body. She also has a history of developmental delay, mild cognitive impairment, adjustment disorder, claustrophobia, recurrent depressive episodes, anxiety disorder, and autism spectrum disorder. Genetic testing confirmed a deletion of exons 4-8 in the *TSC2* gene. At the age of 10, monotherapy with PHT was started, which had such a favorable effect that no more seizures occurred until the age of 19. From the age of 19, she also received everolimus, as a result of which the multi-organ hamartomas regressed significantly. This case shows that epilepsy in TSC is not intractable, that phenytoin could be an option for epilepsy in TSC patients, and that everolimus is very effective in terms of regression of benign tumors.

## Introduction

Tuberous sclerosis complex (TSC) is a rare autosomal dominant multisystem disease that is due to pathogenic loss-of-function variants in the tumor suppressor genes *TSC1* or *TSC2*, which encode the proteins tuberin and hamartin [1,2]. Both proteins inhibit the mTOR (mechanistic target of rapamycin) complex, which is crucial for cell proliferation, growth, and differentiation and is stimulated by various energy sources and hormonal signaling pathways [2]. Mutations in *TSC1* or *TSC2* lead to the target of rapamycin complex 1 (TORC1) hyperactivity, resulting in the formation of hamartomas (benign tumors) that cause abnormal tissue growth and dysfunction in various organs such as the brain, kidneys, heart, lungs, eyes, skin, bones, and teeth [1,2].

In the brain, subependymal giant cell astrocytomas (SEGAs) can occur [1]. Patients develop angiomyolipomas in the kidneys [3]. Rhabdomyomas can develop in the heart [4]. Angiofibromas can be found on the skin. Cerebral involvement in TSC manifests as infantile spasms, antiepileptic seizure medication (ASM)-resistant epilepsy, developmental delays, cognitive impairment, autism spectrum disorders (ASD), and other neurobehavioral manifestations [1]. This complex of abnormalities has been recently referred to as TSC-associated neuropsychiatric disorder (TAND). Recommendations for ASM therapy for TSC-associated seizures include vigabatrin, cannabidiol, everolimus, and the ketogenic diet. TSC is caused in >50% of cases by spontaneous point mutations, deletions, or duplications in *TSC1* or *TSC2*. If it is inherited, transmission is autosomal dominant. Only individual patients with deletions of one or more exons have been reported. A patient with TSC due to a previously unreported deletion of exons 4-8 in *TSC2*, ASM-resistant epilepsy who responded favorably to phenytoin (PHT) and in whom everolimus led to regression of multifocal epilepsy, to the best of our knowledge, has not been previously reported.

## Case presentation

The patient is a 24-year-old female who was diagnosed with TSC at the age of seven years according to the TSC diagnostic criteria 2021 after the onset of epilepsy [5], cerebral imaging suggestive of SEGAs, renal angiomyolipomas, skin angiofibromas, cardiac leiomyomas, retinal hamartomas and deletion of exons 4-8 in the *TSC2* gene detected by multiplex ligation-dependent probe amplification (MLPA) (Table 1). Her medical history was positive for combined developmental delay (first sentences at the age of three), mild cognitive impairment that enabled her to complete elementary school and special education, epilepsy since early childhood, adjustment disorder, claustrophobia, recurrent depressive episodes, anxiety disorder, and ASD. She has been raised by adoptive parents since she was six months old.

**Table 1:** Timeline of events. ASM: anti-seizure mediation, PHT: phenytoin, TSC:  tuberous sclerosis, VPA: valproic acid.

Age (years)	Event	Treatment	Outcome
3	Onset of seizures (psychomotor seizures, absences, auras, tonic-clonic seizures)	None	Recurrent seizures
5	Increase in seizure frequency	VPA	Intractable seizures
7	TSC diagnosed	Various ASMs	Intractable seizures
10	A vagal stimulator was proposed (refused by the father)	PHT	Seizure-free until age 19 yrs
19	PHT reduced, seizures recur	PHT old dosage	Seizure-free since then
19	Everolimus started	Everolimus	Regression of hamartomas, improvement of behavioral abnormalities

The seizures first occurred at the age of three years and manifested themselves semiologically as recurrent auras, complex focal seizures with non-reactivity, immobility, absences, tonic-clonic seizures, and nausea. At the age of eight years, the seizure frequency of staring episodes was five per day. Staring seizures followed by orofacial automatisms occurred at the age of eight years with a frequency of two per month. Generalized tonic-clonic seizures occurred almost every month. The first ASM administered was valproic acid (VPA), which she had been receiving since the age of five. As VPA became ineffective, a number of other ASMs such as vigabatrin, topiramate, carbamazepine, lamotrigine, levetiracetam, zonisamide, sultiam, and rufinamide were tried in the following years, but these were also ineffective. For this reason, an epileptologist recommended implanting a vagus stimulator at the age of 10 to treat the epilepsy, but the adoptive father refused. Therefore, at the age of 10, ASM treatment was switched to PHT in monotherapy, which was so effective that she remained seizure-free until the age of 19. At the age of 19, PHT was reduced because she had already been seizure-free for nine years. As tonic-clonic seizures occurred again during the reduction of PHT, she was put back on the old dose. After restoring the previous PHT dosage, no further seizures occurred until the age of 24.

**Figure 1:**
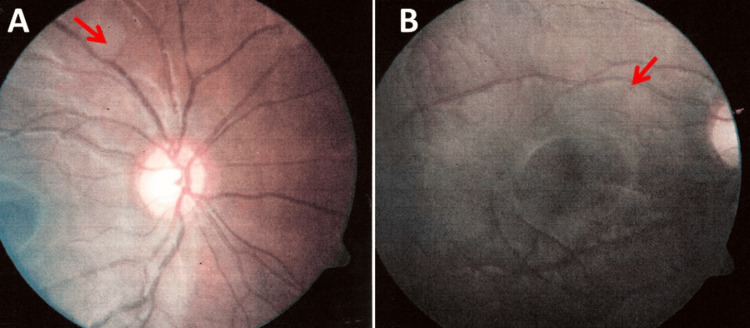
Fundus of the right (A) and left (B) eyes showing the retinal hamartomas

Cerebral magnetic resonance imaging (MRI) at the age of six years showed cortical astrocytomas in the left parietal, left occipital, right temporal, and left temporal distribution. A cerebral MRI at the age of eight years showed giant cell astrocytomas on FLAIR in a subcortical and subependymal distribution bilaterally without significant changes compared to the previous examination. She also noticed black and white spots in both visual fields since the age of 10. An ophthalmologic examination at the age of 11 years revealed hamartomas in the right and left retina (Figure 1). Echocardiography revealed several small leiomyomas in the cardiac septum. Abdominal ultrasonography revealed several hypodense nodular formations in the renal cortex on both sides. A neuropsychological examination at the age of 17 years revealed mainly a perceptual disorder.

**Figure 2:**
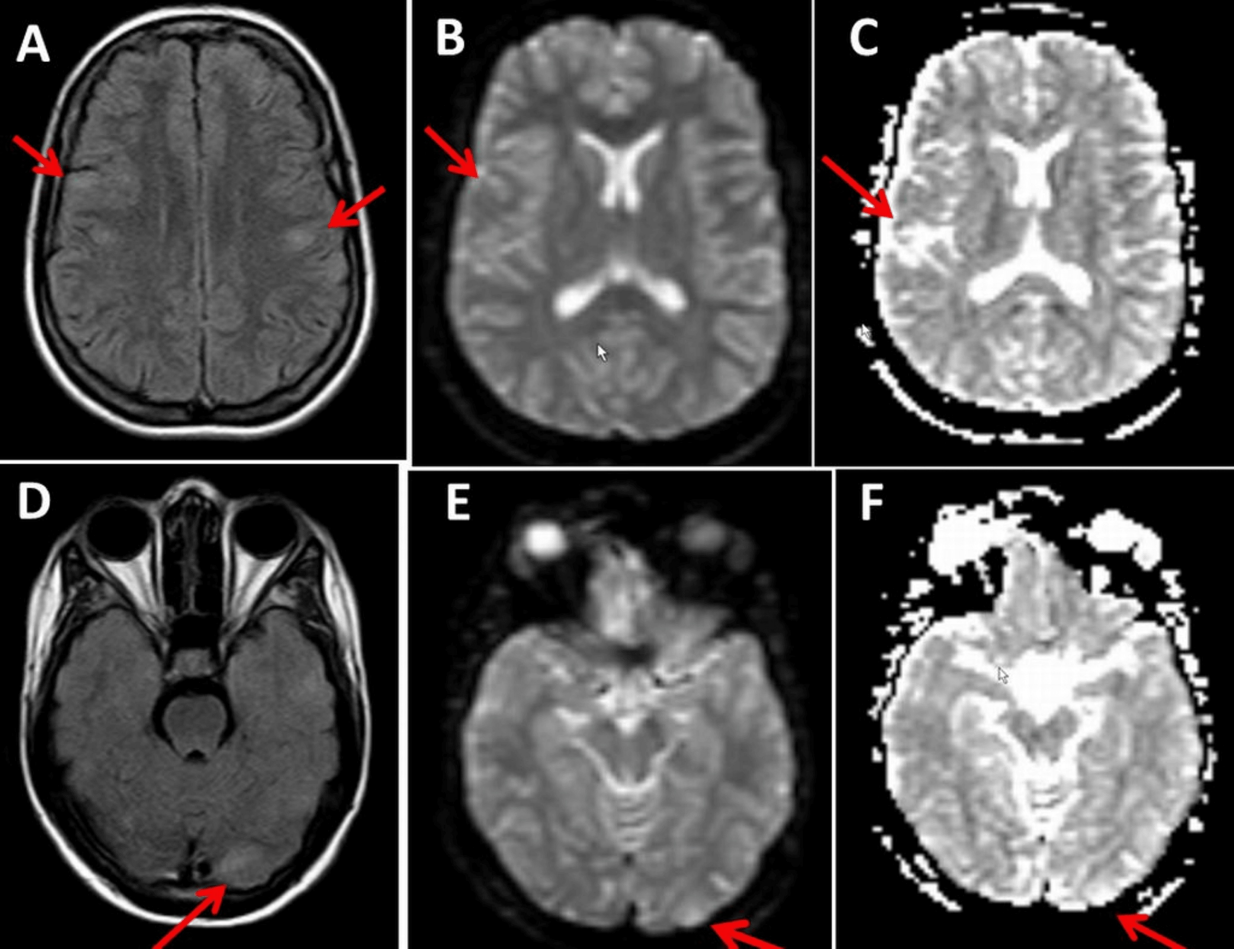
Cerebral MRI images. Cerebral MRI shows vague hyperintense lesions with a diameter of 7 mm in a bilateral parietal distribution subcortically on axial FLAIR (panel A). In addition, a tender hyperintense FLAIR lesion is seen in the left occipital region (panel D), showing mild enhancement in T1 (not shown). These lesions were slightly hyperintense in diffusion-weighted imaging (DWI) (panels B and E) and apparent diffusion coefficient (ADC) images (panels C and F).

She had been receiving everolimus for TSC since the age of 19, which she tolerated without major side effects. Clinical neurologic examination at the age of 24 years revealed an alert, anxious young woman with previous episodes of depression and anxiety, sore neck muscles, scattered angiofibromas over the nose and cheeks, positive pyramidal signs on the right side, exaggerated tendon reflexes on the right side, mild postural tremor bilaterally, weakness when spreading the fingers (M4+) on the left hand and sensory disturbances in the area of distribution of the left ulnar nerve following a traumatic ulnar nerve lesion after surgery for a humerus fracture in 2007, non-fixed inversion position of both feet, slight ataxic stance, rotation of 70 degrees on the Unterberger treadmill test and tendency to fall when walking on a line. MRI of the brain at the age of 20 years showed subcortical flat hyperintense lesions (maximum diameter 7 mm) on FLAIR in the parietal region on both sides and a single discrete hyperintense transcortical FLAIR lesion in the left occipital cortex, which were slightly hyperintense on diffusion-weighted imaging (DWI) but did not enhance (Figure 2). Compared to the previous MRI examinations before the start of treatment with everolimus, the number and size of astrocytomas in the brain had decreased significantly, which was also true for most other extracerebral hamartomas. Blood tests showed slightly elevated liver function parameters but normal phenytoin and everolimus serum levels. Her current medication regimen includes everolimus (7.5 mg/d), phenytoin (425 mg/d), escitalopram (20 mg/d), aripiprazole (5 mg/d), folic acid, and prothipendyl (80 mg) as needed.

## Discussion

The presented patient is interesting for several reasons. First, TSC due to a deletion of exons 4-8 in *TSC2* has not yet been described. The pathophysiological consequences of the deletion of five exons in *TSC2* remain speculative, but it is conceivable that the mutation causes a non-functional protein product that leads to *TORC1* hyperactivity and triggers the proliferation of abnormal tissue. Deletions of different sizes in *TSC2* causing dysregulation of the PI3K/AKTmTOR signaling pathway have been reported previously [6,7,8]. All types of mutations, including nonsense, missense, and frameshift mutations, as well as large rearrangements (deletions/duplications), have been identified in *TSC1* and *TSC2*, but large rearrangements as in the index case are rare [8]. Large rearrangements can easily be overlooked if only sequencing of the *TSC1 *and *TSC2* genes is performed without screening for deletions or duplications using MLPA. In a study of 327 *TSC1 *patients in whom no mutation could be detected by sequencing, deletions were detected by MLPA in eight of them. This study also found that patients with *TSC1 *deletions tend to have a slightly milder phenotype compared to the group of patients with small *TSC1* mutations. Whether the mutation in the index patient was de novo or inherited remains speculative because the index patient's biological parents were not available [9].

Secondly, PHT led to a complete disappearance of seizure activity both clinically and on the EEG. This extraordinary effect started nine years before the start of everolimus treatment, which is why it was attributed exclusively to PHT. Only in single TSC patients has PHT been reported to be ineffective [10]. In a 40-year-old man with chronic kidney disease due to a *TCS2* mutation, PHT adequately suppressed generalized seizures [10]. Interestingly, PHT treatment in this patient was complicated by severe hypocalcemia [11]. PHT was also prescribed as first-line therapy with ASMs in two other TSC patients, but their response to PHT was not reported in detail [12].

Third, everolimus led to a significant reduction or even complete resolution of the harmatomas at each site, as previously reported [13,14]. Whether the administration of everolimus had an additional positive effect on seizure activity remains speculative but is conceivable since the size and number of cerebral lesions regressed significantly, and everolimus was originally approved by the FDA for seizure treatment in TSC [15]. In a study of 64 TSC patients, 45 of whom had epilepsy, 14 (31%) were responders, with seizure frequency decreasing by more than 50% in the last three months of everolimus treatment, and 19 patients (42%) switched their ASM regimen [14]. The administration of everolimus not only improved the imaging findings in the index patient but also the clinical manifestations of TSC, particularly ASD and anxiety disorder. The patient became more proactive, communicated more than before, and experienced fewer episodes of anxiety or panic. This improvement in her psychological corset enabled her to communicate more openly with others and to reverse the previously observed tendency to withdraw. Clinical improvements in the non-epileptic manifestations of TSC with everolimus have also been reported [16,17,18]. In a study of 17 pediatric TSC patients, 14 reported benefits, including a decrease in cardiac rhabdomyoma, improvement in arrhythmia, reduction in SEGA size, and regression of congenital focal lymphedema [16].

In addition to the beneficial effects of PHT and everolimus, some adverse effects were also reported [19]. Ataxia was attributed to PHT because the location of the cerebral lesions did not explain the ataxia, and there was no evidence of polyneuropathy or spinal ataxia on clinical examination. Ataxia is a common side effect of PHT, as previously reported [19]. Other common side effects of PHT include facial edema, anginal chest pain, chills, cough, diarrhea, exertional dyspnea, dysphagia, dysuria, weight gain, acral paresthesias, and sores, ulcers, or white patches on the lips and tongue [19,20]. Although no episodes of hypocalcemia were reported in the index patient, it cannot be ruled out that some of her frequent convulsions were also due to tetany, as TSC has been reported to be complicated by severe hypocalcemia [11]. Adverse effects of everolimus classified according to the common terminology criteria of adverse events (CTCAE) in a study of 17 TCS patients included mild transient stomatitis (two cases), worsening of infantile acne (one case), increases in serum cholesterol and triglycerides (four cases), and changes in serum phosphate levels (two cases), increase in cholinesterase (two cases), transient neutropenia (two cases), transient anemia (one case), transient lymphopenia (one case) and recurrent infections (seven cases) [16]. A rare but life-threatening side effect of everolimus can be pneumonitis [21].

A limitation of the study is that the regression of hamartomas was not well documented on imaging and that the patient was not treated by a psychiatrist. Another limitation is that the biological parents were not clinically or genetically examined.

## Conclusions

This case highlights that epilepsy in TSC is not necessarily intractable, phenytoin can be effective for TSC-associated epilepsy, and everolimus contributes to the regression of benign tumors. Before switching to non-pharmacologic therapy for epilepsy in TSC, PHT should be tried. The identification of the new *TSC2* deletion described here expands the spectrum of mutations that cause TSC.
